# Mechanical Pruning Induces Distinct Metabolic Responses in Slender Spindle-Shaped Apple Orchards

**DOI:** 10.3390/plants14233663

**Published:** 2025-12-01

**Authors:** Juhyeon Park, Youngsuk Lee, Nay Myo Win, Van Giap Do, Jung-Geun Kwon, Seonae Kim, Soon-Il Kwon, Hun-Joong Kweon, In-Kyu Kang

**Affiliations:** 1 Apple Research Center, National Institute of Horticultural and Herbal Science, Rural Development Administration, Daegu 43100, Republic of Korea; kongfo@korea.kr (Y.L.); naymyowin@korea.kr (N.M.W.); giapbio@korea.kr (V.G.D.); dsms0@korea.kr (J.-G.K.); seonaekim@korea.kr (S.K.); topapple@korea.kr (S.-I.K.); kweonhj@korea.kr (H.-J.K.); 2 Department of Horticultural Science, Kyungpook National University, Daegu 41566, Republic of Korea; kangik@knu.ac.kr

**Keywords:** apple, mechanical pruning, transcriptomics, photosynthesis, hormone

## Abstract

Mechanical pruning has emerged as a viable alternative to traditional hand pruning in apple orchards in labor-constrained and aging population workforces. While mechanical pruning reduces labor demand and enhances operational efficiency, their effects on tree physiology and fruit development remain poorly understood. In this study, we examined the physiological and transcriptional responses of apple trees to mechanical pruning (MP) and hand pruning (HP), with a focus on hormone metabolism, photosynthetic activity, and stress adaptation. Pruning treatments were applied in an orchard using a tractor-mounted mechanical pruner and manual shears, and distinct metabolic responses after pruning were assessed over multiple time points using transcriptomic analysis. At 168 h after MP, trees exhibited downregulation of *MdLhcb* genes, indicating a reduction in light harvesting capacity. In addition, *MdDFR*, a key gene in flavonoid biosynthesis, was also downregulated, suggesting a suppression of secondary metabolism and a distinct physiological response to MP. In addition, stress-responsive genes such as *MdNHL3* were rather upregulated, indicating the activation of adaptive signaling networks. Conversely, HP trees showed relatively moderate responses in the same pathways, suggesting pruning method-specific regulatory mechanisms. These findings highlight how pruning methods distinctly influence tree recovery and gene expression dynamics, offering insights into optimizing pruning systems for sustainable and high-quality apple production under labor-constrained conditions.

## 1. Introduction

Stable and high-quality apple production requires comprehensive orchard management practices, including pruning, training, and pest control. Among these, pruning is particularly vital for maintaining tree structure, improving light penetration, reducing pest and disease pressures, and ultimately enhancing fruit quality. Pruning methods can be broadly categorized into hand pruning, semi-mechanized pruning using auxiliary tools, and fully mechanical pruning, each offering distinct advantages and challenges depending on orchard conditions and resource availability. It is reported that the proportion of the population engaged in agriculture declined from 40% in 2000 to 26% in 2022 [1], raising concerns on significant labor shortage, compounded by an aging rural workforce. In South Korea, the rural population has decreased from 2.166 million in 2022 to 2.142 million in 2023, with projections suggesting a further decline to 1.742 million by 2033 [2]. Additionally, the proportion of individuals aged 65 and older is expected to increase from 49.8% in 2022 to 56.2% in 2033, further exacerbating labor shortages in labor-intensive activities such as pruning.

Winter pruning is a crucial orchard management practice that shapes tree structure, regulates fruiting potential, and overall productivity. A labor demand analysis for apple cultivation in 2022 revealed that winter pruning required 21.9 h per 0.1 ha (14.1% of total labor), ranking third after harvesting (49.7 h, 32.0%) and fruit thinning (30.1 h, 19.4%) [3]. In recent years, mechanical pruning has emerged as a promising alternative to traditional hand pruning substantially reducing labor requirements and maintaining consistent pruning quality. Recent studies have examined the effects of mechanical pruning on apple tree growth, yield, and fruit quality, demonstrating that the technique can effectively control canopy density and maintain fruit quality comparable to that of hand pruning, depending on pruning intensity and timing [4]. Studies have shown that mechanical pruning significantly reduces labor time compared to hand pruning [5], without compromising effectiveness. For instance, tractor-mounted pruning system facilitates large-scale pruning operations with minimal manual input, improving overall productivity and orchard sustainability.

In orchards, pruning practice often leads to the reconstruction of tree canopy, which in turn may influence physiological responses to light and hormone metabolism. Optimization of canopy structure can greatly affect photosynthetic efficiency by altering light interception, distribution, and penetration within the canopy [6]. Light is a critical factor in agricultural production, strongly impacting photosynthesis, plant growth, and overall yield [7]. Under different light conditions, plants exhibit differences in photosynthetic rate, electron carriers, and chloroplast structure. In crop production, shading or low-light conditions are recognized as pervasive abiotic stresses that often result from inadequate pruning [8]. In particular, low light condition negatively affects plant physiology, morphology, and vegetative and productive traits. Additionally, trees exposed to low light intensities tend to develop small and thin leaves with reduced chlorophyll content, which reduces stomatal conductance and density, ultimately reducing carbon dioxide assimilation and net photosynthetic rates [9]. Therefore, photosynthetically active radiation (PAR) and solar irradiances are generally measured inside the tree canopies or between the tree intervals to access the receiving amounts of light received by the whole plants for photosynthesis [10,11]. Pruning thus plays an important role not only in optimizing light distribution within the tree canopy but also in balancing the vegetative and reproductive growth of the tree [12]. Moreover, fruit yield and quality are strongly influenced by pruning intensity and light interception [9,13].

Beyond its role in labor management, pruning plays a critical role in tree physiology by influencing key plant hormones that regulate growth and development. These hormones include gibberellins (GA), cytokinins (CK), auxins (IAA), and abscisic acid (ABA), which collectively control processes such as bud growth, cell division, and shoot elongation. Pruning has been shown to increase GA and CK levels, promoting shoot growth, and affecting IAA and ABA, which regulate shoot inhibition and flower bud formation [14]. The magnitude of these hormonal changes depends on pruning method, intensity, and timing. For instance, in peach trees, pruning has been linked to increased auxin concentrations, demonstrating its influence on hormonal metabolism and shoot development [15]. Similarly, in blueberries, top pruning has been found to regulate endogenous hormone levels, significantly impacting flower bud differentiation [16]. These findings underscore the dual role of pruning in both structural and physiological regulation, highlighting its importance in optimizing tree growth and fruit production.

This study aimed to analyze the physiological responses of apple trees and fruit quality under mechanical pruning systems. To investigate the molecular signals involved in metabolic responses induced by mechanical pruning, we developed transcriptome profiles from ‘Fuji’ apple orchard after pruning practices. The key factors responsible for the distinct responses to mechanical pruning were further identified using bioinformatics tools in conjunction with data on canopy structure and photosynthetic efficiency. This study provides an insight into optimization of mechanical pruning strategies for sustainable and high-quality apple production.

## 2. Results and Discussion

### 2.1. RNA-seq Profile Revealed Distinct Tree Responses to Mechanical Pruning in Apple

**Figure 1:**
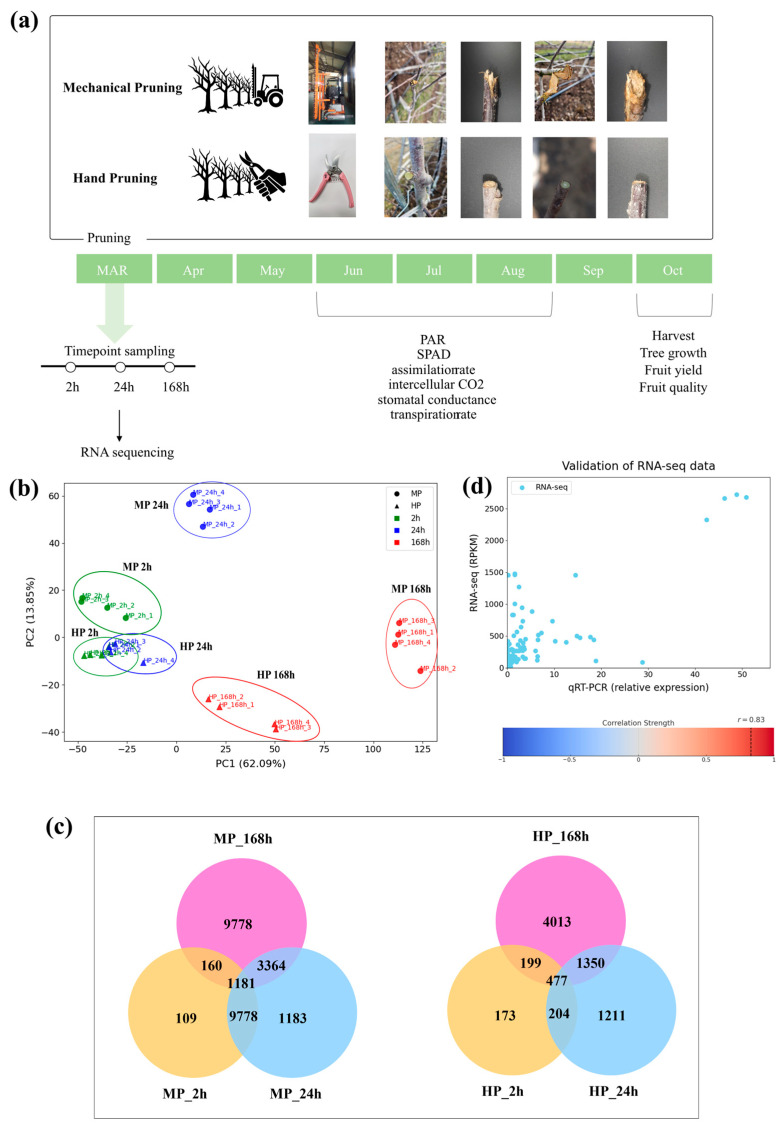
An overview of pruning treatments, sampling schedule, and transcriptome analysis. (**a**) Schematic diagram of the experimental design showing two pruning treatments: mechanical pruning (MP) using a CRF-340 device mounted on a tractor and hand pruning (HP). Photographs illustrate branch appearances after each treatment. (**b**) Principal component analysis (PCA) of RNA-seq data showing the transcriptomic differences among MP and HP treatments over three time points (2 h, 24 h, and 168 h after pruning). (**c**) Venn diagrams showing the number of differentially expressed genes (DEGs) identified at 2 h, 24 h, and 168 h after pruning. (**d**) Correlation analysis between RNA-seq and qRT-PCR data for selected DEGs. A strong correlation (*r* = 0.83, *p* < 0.0001) was obtained between qRT-PCR data and RPKM values from RNA-seq.

To explore the metabolic responses induced by MP, RNA sequencing (RNA-seq) was performed on branch tissues adjacent to those removed via two different pruning treatments: MP and HP across three-time points (2 h, 24 h, and 168 h) (Figure 1a). Compared to HP, MP caused various types of physical damage to branches depending on the blade angle and cutting direction. Although some branches were cleanly severed along the contour of the rotating blades, others had rough or irregular wound surfaces, indicating inconsistent cutting quality. In some cases, incomplete cuts caused tearing when the blades became lodged within branches and were forcibly pulled as the tractor moved forward. These observations suggest that MP can inflict a broad range of wound types, including smooth cuts, rough surfaces, and tearing injuries, particularly in slender, spindle-shaped apple orchards. MP has been reported to alter canopy architecture and wound characteristics relative to HP, potentially eliciting distinct transcriptional and physiological responses [4].

A total of 1981 million clean reads were obtained for 28 libraries from both MP- and HP-tree samples (Table S2). Principal component analysis revealed technical difference between MP and HP. PC1 explained 62.09% of the total variance and distinctly separated the late-stage MP group (MP 168 h) from the others. PC2 explained 13.85% of the variance and further distinguished the early time points of MP (MP 2 h, MP 24 h) (Figure 1b). In contrast, no clear separation was observed between the early time points of HP (HP 2 h, HP 24 h) in the first two PCs. Although MP and HP initially triggered similar transcriptional responses, MP samples exhibited a marked shift after 24 h, whereas HP samples maintained relatively stable patterns.

In total, 17,093 DEGs were identified by comparing samples at each time point with the pre-pruning control, using a threshold of |fold change| ≥ 2 and FDR ≤ 0.05 (Figure 1c and Table S3). The lists of DEGs at each time point are shown in Table S3. The number of DEGs following MP (16,021) was similar to that obtained after HP (16,006). Notably, the number of DEGs in the late-stage MP group (MP 168 h; 14,483 DEGs) was more than twice in the corresponding HP group (HP 168 h; 6039 DEGs), suggesting different physiological responses between the two pruning methods.

To validate the RNA-seq results, qRT-PCR performed on 17 target genes randomly selected from the list of 17,093 pooled DEGs of both pruning treatments. The result showed a strong correlation (*r* = 0.83, *p* < 0.0001) between qRT-PCR data and RPKM values from RNA-seq (Figure 1d, Table S4). These results confirm the reliability of the transcriptome analysis across different time points and pruning methods.

### 2.2. Gene Ontology and Network Analyses Revealed Hormone- and Light-Related Transcriptional Responses to Mechanical Pruning

To investigate the distinct post-pruning metabolic responses induced by MP, gene ontology enrichment analysis was performed on the 17,093 DEGs (Table S5). Among the biological process categories, the most significantly enriched GO terms included light response (GO: 0009642), photosynthesis (GO: 0015979), secondary metabolite biosynthesis (GO: 0019748), responses to stress (GO: 0006950; biotic stress GO: 0009595; abiotic stress GO: 0009628), phytohormone signaling (GA ethylene GO: 0009723; abscisic acid GO: 0009737; jasmonic acid GO: 0071395), starch metabolism (GO: 0005982), and nitrogen transport (GO: 0071705).

**Figure 2:**
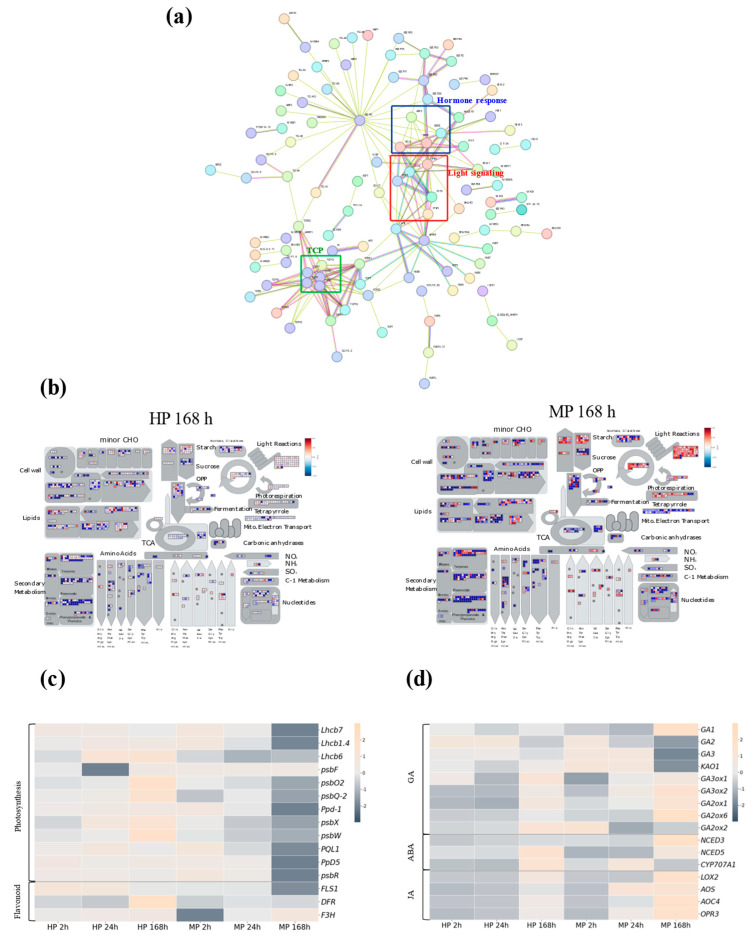
Transcriptomic features and metabolic responses associated with pruning treatments. (**a**) Protein–protein interaction (PPI) network of 192 transcription factors identified from homologous differentially expressed genes (DEGs) in *Arabidopsis* under high-confidence criteria. The network was generated based on STRING analysis, highlighting transcription factor groups related to TCP (green), light signaling (red), and hormone responsive (blue). (**b**) MapMan metabolic overview of DEGs related to primary and secondary metabolic pathways under hand pruning (left) and mechanical pruning (right) at 168 h after pruning. Red and blue colors indicate upregulation and downregulation, respectively. (**c**,**d**) Heatmaps of selected genes involved in (**c**) photosynthesis and flavonoid biosynthesis, and (**d**) hormone metabolism and signaling (GA, ABA, and JA). Differential expression patterns across pruning treatments and time points are displayed for each category.

To visualize the metabolic pathways associated with post-MP responses in apple, we identified 8488 putative *Arabidopsis* orthologs among the 17,093 DEGs (Table S6). A protein–protein interaction (PPI) network of 192 transcription factors was constructed using the STRING database v12.0 with a high-confidence interaction score threshold (≥0.700). These transcription factors were selected based on enrichment analysis of approximately 8000 homologous DEGs mapped to *Arabidopsis thaliana*. The resulting network comprised 106 nodes and 372 edges (Figure 2a). Several highly interconnected modules were identified, notably those involving transcription factors associated with light signaling (e.g., *MdHY5*, *MdPIF3*, *MdFHY3*) and abiotic stress responses (e.g., *MdABF3*, *MdBZIP5*, *MdBZIP63*). The TEOSINTE BRANCHED 1, CYCLOIDEA, and PROLIFERATING CELL FACTOR 1/2 family formed a distinct subnetwork, indicating potential co-regulatory roles in cell growth and developmental processes. Overall, these results suggest a coordinated transcriptional regulatory network that integrates light perception, stress adaptation, and growth regulation in response to MP.

### 2.3. Mechanical Pruning Modulated Light-Harvesting, Hormone Metabolism, and Stress-Associated Pathways in Apple Branches

Metabolic pathway analysis revealed that MP 168 h induced broader and more intense transcriptional changes across multiple metabolic processes, including amino acid metabolism, secondary metabolism, and light reactions, compared to HP 168 h, which exhibited relatively limited transcriptional changes, indicating a more stable metabolic state (Figure 2b).

Genes related to light harvesting and photosynthetic electron transport showed distinct regulation between pruning methods. In MP trees, several *MdLhcb* genes and PSII subunit genes were consistently downregulated across all time points, with the strongest suppression at 168 h. Although downregulation of *MdLhcb* genes can sometimes reflect a photoacclimation response under increased light availability, our physiological measurements do not support this interpretation. MP trees exhibited lower PAR and reduced photosynthetic rates compared with HP trees, indicating that the light environment did not improve after mechanical pruning. Previous studies have reported that physical or wounding stress can reduce the expression of *Lhcb* genes [17], supporting our interpretation that the suppression of *MdLhcb1* and *MdLhcb7* is more likely attributable to stress-induced inhibition of light-harvesting capacity rather than acclimation to higher light. Overall, these results suggest that MP imposes stronger physiological constraints on photosynthetic function than HP (Figure 2c).

Flavonoid-related genes also showed distinct patterns between pruning methods. In MP trees, genes in this pathway displayed an overall downregulation at 2 h, with *MdF3H* showing the most pronounced decrease. Although partial recovery was observed at later time points, both *MdFLS1* and *MdDFR* remained lower than in HP trees at 168 h, indicating a sustained attenuation of the flavonoid biosynthetic pathway under MP.

GA-related genes also showed distinct temporal regulation between treatments. In HP, GA biosynthetic genes exhibited a delayed recovery, whereas in MP, they were reactivated earlier, indicating a faster adjustment of GA metabolism after MP. Notably, GA deactivation genes such as *MdGA2ox1* were upregulated under MP 168 h, suggesting enhanced GA degradation and feedback regulation of GA homeostasis. (Figure 2d). ABA metabolism was also affected by pruning. *MdNCED3* was upregulated under MP 168 h, whereas *MdCYP707A1*, involved in ABA catabolism, was upregulated at earlier time points. JA biosynthesis genes (*MdAOC4*, *MdLOX2*, *MdOPR3*) were strongly upregulated by MP, consistent with previous findings that LOX-family genes are rapidly activated immediately after wounding [18], whereas HP triggered weaker responses in these hormone-related genes (Figure 2d).

Together, these results demonstrate that MP causes greater and more sustained suppression of light-harvesting and PSII-related gene expression, accompanied by pronounced hormonal changes and altered secondary metabolic responses, reflecting distinct physiological adjustments to HP.

### 2.4. Pruning Methods Affected Canopy Light Distribution and Chlorophyll Levels in Apple Trees

GO term enrichment results suggested that the MP induced responses may be related to chloroplast, showing the enrichment of photosynthetic membrane (thylakoid)-related terms (GO: 0009535, GO: 0042651) (Table S5).

PAR was measured to evaluate the sunlight distribution inside tree canopies. In this study, PAR was observed highest in June and steadily decreased from June to August due to the increased development of vegetative organs of the trees (Table 1). Overall, the PAR values were significantly higher in HP trees than in MP trees from June to August. The SPAD index, which reflects chlorophyll concentration in leaves, steadily increased in both pruning treatments. This trend may be due to the increased chlorophyll content and leaf maturity. Although the SPAD values did not differ significantly in June, higher values were recorded in HP trees than in MP trees in July and August. The higher PAR values observed in HP trees indicate more effective sunlight distribution throughout the plant canopy. Additionally, the higher SPAD values indicated that the leaves from HP trees had a greater chlorophyll concentration and better photosynthetic capacity. These findings align with Mika et al. [19], who reported that MP trees received less solar radiations and had poorer light distributions than HP trees. The higher SPAD values observed in HP trees may be attributed to improved light penetration to the entire tree or specific sections of it.

**Figure 3:**
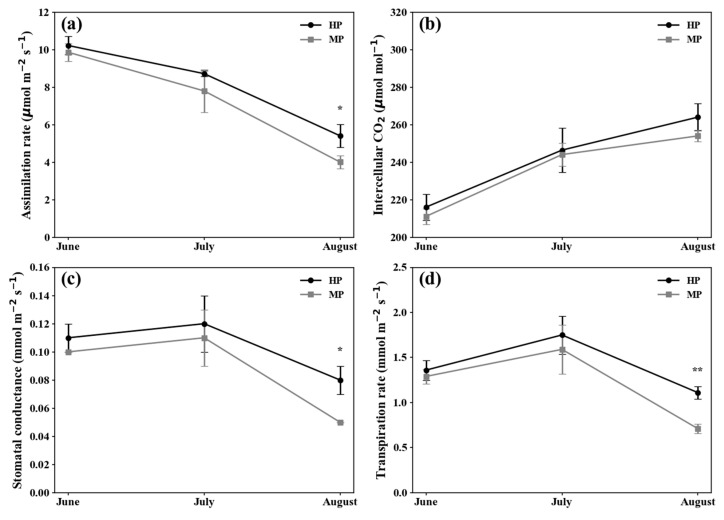
Seasonal changes in photosynthetic and gas exchange parameters under different pruning methods. (**a**) Assimilation rate (*P*_n_). (**b**) Intercellular CO_2_ concentration (*C*_i_). (**c**) Stomatal conductance to water vapor (*G*_s_). (**d**) Transpiration rate (*E*). There were significant differences in *P_n_*, *G_s_*, *E* values between two pruning methods in August. Asterisks indicate statistical significance (*p* < 0.05 for *; *p* < 0.01 for **).

Although higher photosynthetic parameters such as *P*_n_, *C*_i_, *G*_s_, and transpiration rate were recorded in the leaves of HP trees, the results were not statistically different compared to those of MP trees, especially in the months of June and July (Figure 3). Until August, *C*_i_ values did not differ statistically between the two pruning treatments. Bhusal et al. [20] observed a decline in *P*_n_ and *G*_s_ in ‘Fuji’ apple tree (particularly from August until harvest), which has been associated with leaf aging and a reduction in the concentration of enzymes involved in photosynthesis. Additionally, the reduction in *G*_s_ may contribute to the decline in *P*_n_ [21]. In ‘Arisoo’ apples, the non-significant differences of *P*_n_ and *G*_s_ values were observed in the MP trees compared to that of HP trees, while PAR was higher in the HP trees than that of MP trees [4]. The photosynthesis rate in plants is not only related with the intensity of PAR, but also affected by the efficiency of light quantum chemistry [22,23]. However, in this study, higher *P*_n_ values were still observed in the HP compared to that in MP in August. Therefore, it should be noted that the variations in the *P*_n_ and *G*_s_ values might be varied depending on the tree species, leaf maturity, leaf chlorophyll levels, and environmental conditions of the orchard.

### 2.5. Negatively Correlated Genes Revealed Antagonistic Transcriptional Responses Between Pruning Methods in Apple Trees

Given the distinct gene enrichment patterns observed in the post-pruning responses to MP and HP in the apple orchard, we hypothesized that specific genes may be mainly involved in metabolic responses particularly induced by MP and that their differential expression patterns would remain consistent across post-pruning time points.

**Figure 4:**
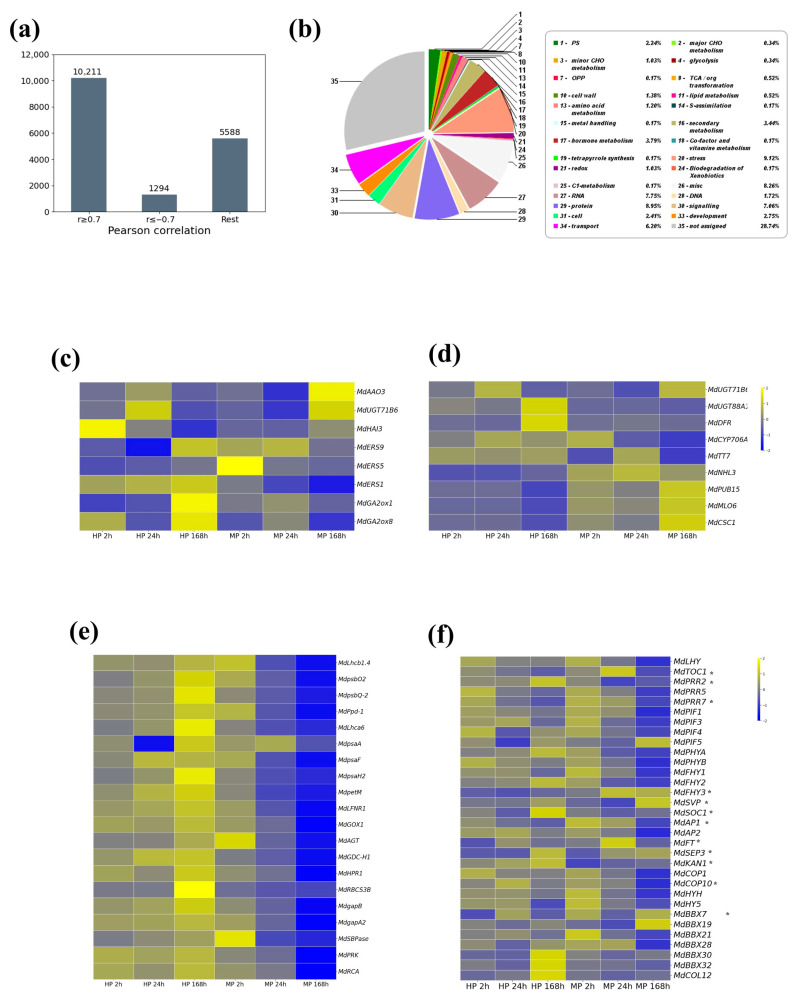
Investigation of 1294 negatively correlated DEGs involved in the metabolic responses to mechanical pruning. (**a**) Distribution of genes based on Pearson correlation between pruning treatments. Genes were classified into three groups according to the correlation coefficients: highly positive (*r* ≥ 0.7), highly negative (*r* ≤ −0.7), and the rest. Among them, 1294 DEGs with strong negative correlation (r ≤ −0.7) were selected for further metabolic pathway analysis. (**b**) Functional categorization of negatively correlated DEGs (*r* ≤ −0.7). Classification of the 1294 negatively correlated DEGs (neg-cor DEGs) using Mercator v3.6 based on DNA sequence homology. (**c**–**f**) Heatmaps of selected genes involved in (**c**) hormone signaling; (**d**) flavonoid biosynthesis and stress responses; (**e**) photosynthesis; (**f**) light signaling, circadian clock regulation, and floral transition including B-box (BBX) genes. Differential expression patterns across pruning treatments and time points are displayed for each category. Asterisks (*) indicates genes that were not found as differentially expressed in our transcriptome data.

To explore this further, we conducted Pearson’s correlation analysis of the transcriptome dataset. Among the 17,093 DEGs pooled from both pruning groups, 3521 (19.3%) genes exhibited negative correlation between the two groups, whereas 13,572 (80.7%) showed positive correlation (Figure 4a, Table S7). From the negatively correlated set, we identified 1294 DEGs (hereafter referred to as neg-cor DEGs) with a strong negative correlation (*r* ≤ −0.7). These neg-cor DEGs were considered of particular interest because they represent antagonistic gene expression patterns between the two pruning groups and may reflect specific MP-induced responses in apple trees. Based on DNA sequence homology to *Arabidopsis*, the 1294 neg-cor DEGs were grouped into functionally important categories, such as photosynthesis (Bin 1), CHO metabolism (Bins 2, 3), cell wall (Bin 10), secondary metabolism (Bin 16), hormone-related processes (Bin 17), stress response (Bin 20), and RNA-related functions (Bin 27) (Figure 4b; Table S8).

### 2.6. Pruning-Induced Transcriptome Changes Revealed Differential Regulation of Photosynthesis, Hormone Signaling, and Light-Responsive Pathways in Apple Branches

MP induced extensive transcriptional reprogramming, particularly affecting hormone metabolism, photosynthesis, and light-responsive pathways. ABA-associated metabolic and signaling activity displayed a transient activation under HP but a delayed, stronger activation under MP. This divergence suggests that the two pruning methods elicit distinct wound environments that differentially shape ABA-mediated stress adjustment processes. Whereas HP promoted a gradual enhancement of ethylene-related signaling, MP consistently suppressed it. This pattern indicates that ethylene-mediated responses may be more engaged following HP, while mechanical pruning attenuates ethylene signaling. *GA2ox* genes are known to function as key regulators of gibberellin (GA) deactivation across higher plants [24]. We found that both *MdGA2ox1* and *MdGA2ox8* were strongly upregulated at HP 168 h. In contrast, their progressive downregulation under MP indicates a distinct hormonal adjustment pattern associated with MP (Figure 4c).

Flavonoid pathway genes, such as *MdDFR* and *MdUGT88A1,* were again found to be strongly upregulated under HP 168 h, whereas MP elicited weaker or delayed responses. Notably, *MdDFR* and *MdTT7* were downregulated at MP 168 h, indicating a pruning-type-specific regulation of flavonoid-associated metabolism (Figure 4d). The gene *MdUGT71B6*, a glycosyltransferase involved in ABA conjugation secondary metabolism, showed time-dependent changes under both pruning treatments, suggesting dynamic regulation of ABA-related and secondary metabolic pathways. In addition to flavonoid-related genes, several stress- and signaling-associated genes exhibited contrasting responses between the two pruning treatments. Notably, *MdNHL3*, *MdMLO6*, *MdCSC1*, and *MdRUB15* showed consistent upregulation under MP, whereas their transcript levels were downregulated under HP. This pattern is likely linked to the greater physical injury caused by MP, such as branch tearing and exposed wound surfaces. Among these genes, *MdNHL3* provides additional context, as *NHL3* overexpression in *Arabidopsis* has been associated with enhanced defense capacity [25].

Photosynthesis-related processes were broadly suppressed under MP, especially at 168 h. Genes encoding components of the light-harvesting complexes and photosynthetic electron transport, including *MdLhcb*, *MdpsbO*, and *MdpsbQ*, were strongly downregulated, while HP trees exhibited recovery at later stages (Figure 4c). Light-harvesting complex proteins, including *Lhcb* family members, are involved in stress-responsive regulation of photosynthetic processes [26]. Thus, the altered expression of *MdLhcb* genes observed under MP may be associated with wound-related stress responses. Notably, *MdpsaA*, a *PSI* core gene, remained suppressed in MP trees but gradually recovered in HP trees, suggesting persistent photoinhibition under MP. The sustained suppression of this gene under MP likely reflects impaired PSI function as a response to pruning-induced stress. Several Calvin cycle genes, including *MdRBCS3B*, *MdgapA*, *MdSBPase*, and *MdPRK*, were suppressed at MP 168 h, indicating inhibition of carbon assimilation. In contrast, HP 168 h samples maintained stable or increased transcript levels, suggesting recovery of photosynthetic capacity (Figure 4c).

To further explore transcriptome data, we investigated the expression patterns of genes associated with light perception, circadian clock, and floral transition. CONSTANS (CO) is a key integrator of light signaling, circadian clock, and flowering regulation [27]. At MP 168 h, genes involved in light perception (*MdPIF1*, *MdPIF3*, *MdPIF4*, *MdPHYA*, *MdPHYB*, *MdFHY1*, and *MdFHY2*) and circadian regulation (*MdTOC1*, *MdLHY*, *MdPRR2*, *MdPRR5*, and *MdPRR7*) were downregulated, suggesting a suppression of light signaling and circadian rhythms. Correspondingly, floral transition genes (*MdSOC1*, *MdAP1*, and *MdAP2*) also exhibited decreased transcript levels, indicating that MP may delay flowering processes. In apple, B-box (BBX) genes are known to regulate light responses [28]. Among the 17,903 DEGs identified, *MdBBX30* and *MdBBX32* were significantly downregulated, whereas *MdBBX7* and *MdBBX19* were upregulated, suggesting that MP and HP treatments differentially modulate light signaling pathways (Figure 4f).

GO enrichment analysis further revealed significant enrichment of the biological process term “response to light intensity” (GO:0009642; FDR 0.05), which included 305 genes. This result supports the transcriptional changes observed in light perception and circadian signaling genes, such as *MdPIF1*, *MdPHYA*, and *MdBBX30*, all of which were downregulated under MP. These findings suggest that MP reprograms light-responsive pathways in apple trees, potentially influencing both flowering time and photosynthetic regulation.

### 2.7. Pruning Method Affected Fruit Quality Traits Without Significantly Altering Tree Growth or Yield in Apple

Flower set density per tree was recorded during the flower blooming period, but no significant differences were observed between the two pruning treatments (Table 2). Flower set density is an important factor for plant yield because a reduction in flower set density can negatively affect orchard productivity and profitability [29]. The lack of reduction in flower set density suggests that MP does not reduce orchard yield and productivity.

The height and canopy width of the tree and shoot length did not differ significantly between the two pruning treatments (Table 2). Although shoot length was slightly greater in MP trees, this difference was not statistically significant. Previous studies have reported that MP may promote excessive shoot growth [12,19], which may explain the slightly longer shoots observed under MP in this study.

Fruit quality parameters such as fruit yield, fruit per tree, fruit weight, fruit shape (length: diameter ratio of fruit), flesh firmness, soluble solids content (SSC), titratable acidity (TA), and fruit color index were measured at harvest (Table 3). Productivity (yield and fruits per tree) was not significantly different between both pruning treatments. However, HP significantly improved the average fruit weight, L/D ratio, and color index.

Pruning is known to influence tree growth and yield [30]. Lin et al. [9] reported a positive correlation between leaf *P*_n_ and fruit weight, suggesting that increased leaf photosynthesis contributes to heavier fruit. Additionally, ref. [12] found that MP often results in poor fruit coloration due to suboptimal light distribution within the tree canopy. Consistent with these findings, the improved fruit weight and color observed in HP trees may be primarily attributed to better light penetration and enhanced leaf photosynthesis activities in plants. However, the values of flesh firmness, SSC, and TA were not significantly different between the pruning treatments (Table 3). These results are consistent with previous observation by He and Schupp [12], who reported that non-selective MP had limited ability to improve the quality of fruit.

## 3. Materials and Methods

### 3.1. Experimental Design and Pruning Practice

This study was conducted on five-year-old apple trees (*Malus* × *domestica* Borkh cv. Fuji), planted with a spacing of 3 m between rows and 1 m within rows, and trained in the Slender Spindle training system.

Pruning treatments were divided into mechanical pruning (MP) and hand pruning (HP) groups (Figure 1a). MP was performed using a CRF-340 mechanical pruner (Rinieri, Forlí, Italy) mounted on a tractor that moved along the row to trim branches. The machine was a trimmer-type device with fixed blades that moved vertically to cut branches. Cutting bar was slightly tilted by approximately 20°, allowing partial pruning of the upper canopy as well. Branches were pruned back to approximately 40 cm from the trunk. Ten individual trees were selected for each pruning treatment. HP was performed using horticultural pruning shears, following the methods as described by Robinson et al. [31]. Thick or unnecessary branches were removed using thinning cuts, and canopy width was adjusted to prevent overlapping with adjacent trees. Additionally, unnecessary branches were pruned to retain three to five flower buds per branch.

### 3.2. Transcriptomics

#### 3.2.1. RNA Extraction and Sequencing

Total RNA was extracted from branch tissues of ‘Fuji’ slender spindle-shaped apple trees using a modified CTAB method [32]. For sampling, branches closed to the tissues cut during pruning were collected at three time points: 2 h, 24 h, 168 h post-pruning. Ten biological replicates were pooled for each sampling point. RNA quality was assessed using Agilent 2100 Bioanalyzer (Palo Alto, CA, USA), and samples with a RNA Integrity Number (RIN) of 7 or higher were submitted to UnGENE Company (Seoul, Republic of Korea) for sequencing. There were four replicates per each sampling group for RNA sequencing. RNA-seq libraries were prepared using the Illumina TruSeq Stranded mRNA sample preparation kit (San Diego, CA, USA), and a total of 28 libraries were constructed and sequenced on the Illumina Novaseq 6000 platform (San Diego, CA, USA) using 2 × 101 bp paired-end (PE) reads.

#### 3.2.2. RNA-seq Read Processing, Mapping, and Expression Data Analysis

Paired-end (PE) reads that met the following criteria were retained for further analysis: a minimum quality score of 0.05 (equivalent to Phred Q13), no more than two ambiguous nucleotides, and a minimum length of 75 bp. The trimmed reads were mapped to the apple reference genome GDDH13v1.1 [33], which includes 51,000 genes, comprising 45,116 protein-coding genes and various noncoding RNAs, using analysis tool in the CLC Genomics Workbench 21 (QIAGEN, Hilden, Germany). Mapping parameters were set to a minimum length fraction of 0.8 and a minimum similarity fraction of 0.8. Gene expression levels were normalized as reads per kilobase per million (RPKM). Differentially expressed genes (DEGs) were identified based on a |fold change| of ≥1.5 and a false discovery rate (FDR) of ≤0.05 to compare MP and HP trees at three time points. Gene ontology (GO) term enrichment analysis was performed using the agriGO tool [34]. The metabolic pathway enrichment analysis was conducted using the MapMan software database [35].

### 3.3. Quantitative Reverse Transcription PCR (qRT-PCR)

First-strand complementary DNA (cDNA) was synthesized from 1 μg of total RNA using an oligo dT primer and Transcriptor Reverse Transcriptase (Roche, Penzberg, Germany). Quantitative real-time PCR (qRT-PCR) was performed using the LightCycler 480 SYBR Green I Master Mix (Roche, Germany) on a Roche LightCycler^®^ 480 system (Basel, Switzerland). A 20-fold diluted cDNA was used as the template (5 μL) in a total reaction volume of 20 μL. Each sample was analyzed with four to six technical replicates. The PCR conditions consisted of an initial denaturation at 95 °C for 5 min, followed by 45 cycles of denaturation at 95 °C for 10 s, annealing at 65 °C for 15 s, and extension at 72 °C for 12 s. A melt curve analysis was performed at the end of the reaction to confirm the amplification specificity of a single product. Primers were designed using Primer-BLAST (http://www.ncbi.nlm.nih.gov (accessed on 15 May 2024)) to span an intron when possible, with an expected amplicon size of 100–150 bp (Table S1). *MdACTIN17* was used as the reference gene. Primer efficiencies and relative transcript abundance were determined using the Roche LightCycler 480 software E-Method [36].

### 3.4. Measurements of Sunlight Intensity and Soil–Plant Analysis Development (SPAD)

Photosynthetically active radiation (PAR) was measured at three locations, both sides and the inner canopy, of each apple tree at approximately 1 m height above the ground, following the method described by Win et al. [37]. PAR was separately measured at the upper, middle, and lower canopy positions, and the average value was calculated for each tree. The measurements were conducted at midday on sunny days during the summer months (June, July, and August) using a light sensor meter (3415FX, Spectrum Technologies Inc., Aurora, IL, USA). SPAD values were recorded on three randomly selected mature leaves located within the inner canopy of each apple tree using a chlorophyll meter (SPAD-502 Plus, Konica Minolta, Tokyo, Japan). For SPAD measurements, both young and mature leaves were sampled at approximately 100 cm above the ground.

### 3.5. Measurements of Photosynthetic Parameters

Photosynthetic parameters such as assimilation rate (*P*_n_), intercellular CO_2_ concentration (*C*_i_), stomatal conductance (*G*_s_), and transpiration rate were measured between 9:00 a.m. and 12:00 p.m. on clear days in June, July, and August of 2024. For each apple tree, six fully expanded mature leaves, (typically the 5th or 6th leaf from the tip of a growth shoot), located on the outer canopy on both sides of the tree were used for measurement. Measurements were performed using an open gas exchange system (LI-COR 6400, Li-COR Inc., Lincoln, NE, USA) equipped with a leaf chamber and red-blue LED light source. The photosynthetic rate and stomatal conductance were measured using a portable photosynthesis system. The measurement conditions were set to a light intensity of 700 μmolm^−2^s^−1^ and a CO_2_ concentration of 400 μmolmol^−1^.

### 3.6. Measurements of Tree Growth Characteristics

Tree growth characteristics, such as shoot length, tree width and length, and tree height, were measured after harvest. Shoot length was measured in fifteen shoots of each apple tree (150 shoots per treatment). Flower set density was first recorded for each tree during the blooming period on 18 April 2024. Shoot length, tree width, and tree length were measured using a measuring tape (Digikey Co., Suwon, Republic of Korea) and expressed in cm. Tree height was measured using a tower ruler (Levenhuk Co., Praha, Czech) and also expressed in cm.

### 3.7. Measurements of Fruit Yield and Fruit Quality Parameters

Fruit yield and fruit quality parameters were measured at harvest (26 October 2024). Total fruit weight per tree was measured and recorded as fruit yield (kg/tree). Individual fruit weight was also measured and presented as average fruit weight (g). Fruit length (L) and diameter (D) were measured, and fruit shape was presented as L/D ratio. Fruit color (L*a*b*) was measured using a chroma-meter (CR-400, Konica Minolta, Tokyo, Japan), and the fruit color index was calculated as follows:

$$\mathrm{Fruit}\;\mathrm{color}\;\mathrm{index}\;=\;1000\;\times \;\frac{a*}{\left(L*\;\times \;b*\right)}$$

following the equation used to quantify fruit surface coloration [38]. Flesh firmness was measured at three points per fruit using a firmness tester (TR-327, Forlí, Italy). Soluble solids content (SSC) was measured using a refractometer (PR-201, Atago, Tokyo, Japan). Titratable acidity was determined by titrating fruit juice samples of fruit with 1 N NaOH, following the malic acid reduction method [37]. Fifteen apple fruits per tree (150 apples per treatment) were used for all measurements of fruit quality attributes.

### 3.8. Quantification and Statistical Analyses

Hierarchical clustering of gene expression data was visualized using a heatmap generated with the Pheatmap package in R (https://cran.r-project.org/web/packages/pheatmap/ (accessed on 10 October 2024)). Functional annotation of genes was conducted using the Mercator online tool [39] to identify putative *Arabidopsis* orthologs within the apple transcriptome dataset. For the statistical analysis of PAR, SPAD, photosynthetic parameters, tree growth characteristics, and fruit quality between MP and HP trees, independent *t*-test was performed using SPSS software (Version 26, IBM Corp, Armonk, NY, USA). Additional statistical analyses were performed using Microsoft Excel 2016 and the R programming environment (http://www.R-project.org/ (accessed on 10 October 2024)).

## 4. Conclusions

Our findings provide a molecular framework for understanding the distinct physiological responses triggered by MP and HP in apple trees. They reveal that the coordination of photosynthetic recovery, secondary metabolism, hormonal regulation, and stress response is modulated through a set of key genes. Notably, under MP 168 h, significant transcriptional reprogramming was observed in genes associated with photosynthesis, hormone metabolism, secondary metabolism, and stress responses. Moreover, MP did not remarkably affect tree yield and fruit quality attributes, but it produced small-sized and poor-colored fruits.

The decreased expression of *MdLhcb* indicates a suppression of photosynthetic activity and a reduction in Calvin cycle processes. Concurrently, the downregulation of *MdDFR* suggests a suppression of secondary metabolism, which may lead to reduced pigment accumulation and a weakened stress defense under MP treatment.

**Figure 5:**
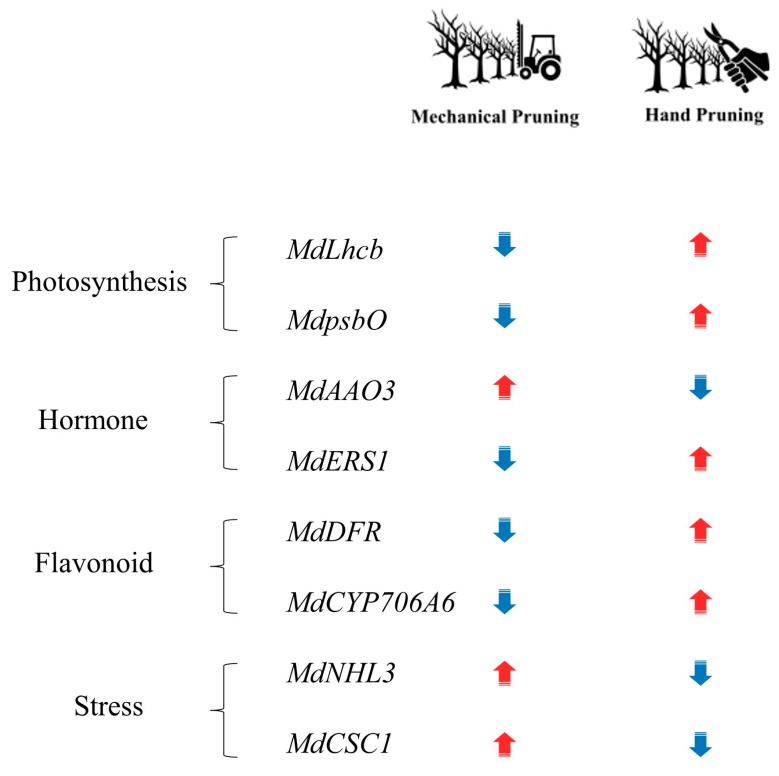
A proposed scheme for molecular signals associated with physiological responses to mechanical pruning in apple orchards. Key gene expression responses to mechanical pruning (MP) and hand pruning (HP) treatments are shown including photosynthesis, hormone signaling, secondary metabolism, and stress responses. Upward and downward arrows indicate upregulation and downregulation, respectively.

Additionally, the involvement of stress-responsive ion channels such as *MdCSC1* is indicative of the activation of early signal transduction processes, highlighting their critical role in initiating environmental adaptation in response to pruning-induced stress. The contrasting expression patterns of key genes related to photosynthesis, hormone regulation, flavonoid metabolism, and stress signaling further illustrate the distinct molecular responses between mechanical and hand pruning (Figure 5). Collectively, MP induced stronger and more persistent transcriptional reprogramming than HP, indicating that MP triggers pronounced physiological adjustments involving photosynthetic regulation, hormone metabolism, and secondary metabolism.

Our detailed understanding of molecular traits induced by MP may contribute for future research in the application of mechanization for the cultivation in apple orchards.

## Figures and Tables

**Table 1:** Influence of hand and mechanical pruning on the photosynthetically active radiation (PAR) and SPAD in ‘Fuji’ apple trees in June, July, and August of 2024.

Treatments	PAR (µmol m^−2^ s^−1^)			SPAD (µmol m^−2^)		
	June	July	August	June	July	August
Hand pruning	506.56 ± 84.43	337.60 ± 2.92	258.07 ± 6.19	52.80 ± 1.39	56.09 ± 0.90	59.26 ± 0.23
Mechanical pruning	267.91 ± 27.31	158.73 ± 18.67	143.98 ± 11.79	51.33 ± 0.76	53.44 ± 0.55	57.34 ± 0.13
Significance	*	**	**	ns	*	**

Data are presented as mean ± standard error (*n* = 3), and each replicate consisted of five apple trees. Mean values between two pruning treatments are compared using a *t*-test at * *p* < 0.05 and ** *p* < 0.01 significant levels. ns: non-significant.

**Table 2:** Influence of hand and mechanical pruning on the flower set density, shoot length, truck cross-sectional area (TCSA), tree height, and tree width of ‘Fuji’ apple trees.

Treatments	Flower Set Density (Set/Tree)	Shoot Length (cm)	Tree Height (cm)	Canopy Width (cm)	
				Length	Diameter
Hand pruning	135.17 ± 8.30	32.30 ± 3.33	410.00 ± 5.77	160.33 ± 15.90	207.67 ± 15.90
Mechanical pruning	132.58 ± 18.21	38.63 ± 2.62	383.33 ± 27.28	149.67 ± 6.17	173.00 ± 7.37
Significance	ns	ns	ns	ns	*

Data are presented as mean ± standard error (*n* = 3), and each replicate consisted of five apple trees. Mean values between two pruning treatments were compared using a *t*-test, and statistical significance was determined at * *p* < 0.05. ns: non-significant.

**Table 3:** Influence of hand and mechanical pruning on the fruit yield, fruit weight, fruit shape (length/diameter ratio of fruit), fruit color index, flesh firmness, soluble solids content (SSC), titratable acidity (TA), and starch pattern index (SPI) of ‘Fuji’ apple trees at harvest.

**Treatments**	**Yield** **(kg/Tree)**	**No. of Fruit** **(Fruit/Tree)**	**Fruit Weight** **(g)**	**Fruit Shape** **(L/D Ratio)**
Hand pruning	19.43 ± 1.22	71.00 ± 4.57	274.12 ± 5.54	0.87 ± 0.00
Mechanical pruning	17.39 ± 1.10	71.85 ± 4.35	242.22 ± 5.78	0.84 ± 0.01
Significance	ns	ns	**	*
	**Flesh Firmness** **(N)**	**SSC** **(°Brix)**	**TA** **(%)**	**Fruit Color Index**
Hand pruning	53.33 ± 0.99	11.79 ± 0.58	0.34 ± 0.01	3.71 ± 0.35
Mechanical pruning	53.81 ± 0.61	11.70 ± 0.19	0.34 ± 0.00	2.74 ± 0.39
Significance	ns	ns	ns	*

Data are presented as mean ± standard error (*n* = 3), and each replicate consisted of thirty apple fruits. Mean values between two pruning treatments were compared using a *t*-test at * *p* < 0.05 and ** *p* < 0.01 significant levels. ns: non-significant.

## Data Availability

All data generated or analyzed during this study are included in this published article and its Supplementary Information files.

## Supplementary Materials

The following supporting information can be downloaded at: https://www.mdpi.com/article/10.3390/plants14233663/s1, Table S1: Primer sequences of gene used for qRT-PCR. Table S2: Summary statistics of RNA-seq data. Table S3: DEGs at each stage and the expression level (RPKM) of 17,093 DEGs obtained from mechanical pruning (MP) and hand pruning (HP) samples. Table S4: qRT-PCR results compared against RNA-seq data. Table S5: GO enrichment analysis of 17,093 DEGs. Table S6: Enrichment of orthologs of Arabidopsis in 17,093 DEGs to protein–protein interaction (PPI) network. Table S7: Pearsons’s correlation analysis of the total 17,093 DEGs between mechanical and hand pruning samples. Table S8: Functional annotation of 1294 negatively correlated DEGs identified in this study.
